# The role of IL-33 and mast cells in allergy and inflammation

**DOI:** 10.1186/s13601-015-0076-5

**Published:** 2015-09-29

**Authors:** Rohit Saluja, Mahejibin Khan, Martin K. Church, Marcus Maurer

**Affiliations:** Department of Dermatology and Allergy, Allergie-Centrum-Charité, Charité-Universitätsmedizin Berlin, Berlin, Germany; Central Food Technological Research Institute-Resource Centre, Lucknow, India; Department of Biochemistry, All India Institute of Medical Sciences, Bhopal, Madhya Pradesh 462024 India; Ramalingaswami Fellow, Department of Biotechnology, Government of India, New Delhi, India

**Keywords:** Mast cell, IL-33, MyD88, MAP kinases, Allergic asthma, Atopic dermatitis

## Abstract

Interleukin-33 (IL-33) is a member of the interleukin-1 (IL-1) cytokine family. It is preferentially and constitutively expressed in different structural cells such as epithelial cells, endothelial cells, and smooth muscle cells. During necrosis of these cells (after tissue injury or cell damage), the IL-33 that is released may be recognized by different types of immune cells, such as eosinophils, basophils and, especially, mast cells. IL-33 needs the specific receptor ST2 (membrane-bound receptor) and Interleukin-1 receptor accessory protein heterodimer for its binding, which instigates the production of different types of cytokines and chemokines that have crucial roles in the exacerbation of allergic diseases and inflammation. IL-33 and mast cells have been influentially associated to the pathophysiology of allergic diseases and inflammation. IL-33 is a crucial regulator of mast cell functions and might be an attractive therapeutic target for the treatment of allergic and inflammatory diseases. In this review, we summarize the current knowledge regarding the roles of IL-33 and mast cells in the pathogenesis of allergies and inflammation.

## Background

Interleukin-33 is a recently discovered cytokine that belongs to the IL-1 super-family and is mainly expressed by different types of structural cells [1, 2]. IL-33 binds to a specific receptor named T1/ST2 (also known as ST2) that belongs to the toll like receptor (TLR)/IL1R super family [3]. The T1/ST2 receptor forms heterodimer with the IL-1 receptor accessory protein (IL-1RAcP). Both receptors are required for the binding and action of IL-33. ST2 receptor has two major isoforms: a transmembrane form (ST2 or ST2L) and a soluble form (sST2) [2]. The ST2L isoform is mainly expressed on mast cells, basophils, dendritic cells, natural killer cells and Th2 lymphocytes [1–3]. IL-33 is considered an alarm in molecule due to its release after necrosis or tissue damage. In contrast, apoptosis leads to the inactivation of IL-33 because it is cleaved by caspases. Different stimuli such as bacterial, viral, fungal infections and allergen challenges can trigger the release of IL-33. Recent research suggests that IL-33 plays an important role in allergy and inflammation. In this review, we will focus on the recent advancements in the field of IL-33 and its association with mast cells in the context of allergy and inflammation.

## Source of IL-33: expression, release and processing

### Expression of IL-33 in physiological and pathological conditions

Interleukin-33 transcript and protein is widely expressed in different cell types including in cells of both hematopoietic as well as non-hematopoietic origin such as macrophages, dendritic cells, fibroblasts, adipocytes, smooth muscle cells, endothelial cells, bronchial, osteoblast and intestinal epithelial cells [4, 5]. Schmitz et al. [5] demonstrated that IL-33 mRNA is expressed in purified dendritic cells, epithelial cells, activated macrophages and it was also confirmed that its expression was much higher in stomach, lung, brain and skin tissues. A more detailed summary of IL-33 distribution is shown in Table 1.

**Table 1:** Expression of IL-33 in different cells/tissues

Species	Expression	Specific cell/tissue	References
Human	Transcript	Human epithelial cells (A549)	[32]
Human	Transcript	Macrophage	[5]
Human	Transcript and protein	Human adult cardiac fibroblasts (HACF) and human adult cardiac myocytes (HACM)	[68]
Human	Transcript and protein	Airway smooth muscle (ASM), bronchial epithelium	[62]
Human	Transcript and protein	Pancreas	[69]
Human	Protein	Mast cell	[62]
Human	Protein	Endothelial cells and epithelial cells, Lymphoid tissues, keratinocytes and stomach (epithelial cells)	[7]
Human	Protein	Fibroblast	[70]
Human	Protein	Skin	[71]
Mice	Transcript	Macrophage	[5]
Mice	Transcript	Glial cells, astrocytes	[1]
Mice	Transcript	Murine lung epithelial cells (*MLE*-*15)*	[32]
Mice	Transcript	Central nervous system	[4]
Mice	Transcript	Lungs	[32]
Mice	Transcript and protein	Eye and cervical lymph nodes (CLNs)	[72]
Mice	Transcript and protein	Pancreas	[69]
Mice	Protein	Dendritic cells	[73]
Mice	Protein	Alveolar epithelial and endothelial cells	[32]
Mice	Protein	Bronchoalveolar lavages	[32]

Like IL-1α/β and IL-18, IL-33 functions as a transcriptional regulator [6] in high endothelial venules (HEVs), and has been reported to be also expressed within the nuclei of epithelial cells [7]. Wood et al. [8] studied the expression of IL-33, IL-1RL1 and IL-1RAcP gene in human pre-adipocytes and in adipocytes (SGBS cells). Expression of IL-33 has also been detected in epithelial cells, skin, lungs, and gastrointestinal tract [7, 9]. Andronicos et al. [10] established that damage caused by motile gastrointestinal nematode larvae in parasitic infection significantly induced IL-33 mRNA expression in epithelial cells. Later, it was reported that IL-33 also plays an important role in innate immune responses, for example during influenza virus infections in the lungs [11] and in helminth infections in the intestine [12, 13].

Interleukin-33 expression was found to be increased in several pathological conditions such as airway smooth muscle cell [14] and lung epithelial cells of asthmatic patients [9] as well as in airway epithelial cells in COPD patients [15]. Expression of IL-33 was also reported in liver cells (hepatocytes) and in inflamed colon in a mouse model of acute hepatitis [16] and colitis [4], respectively. IL-33 also plays a crucial roles in the initiation as well as amplification of a type 2 response in group 2 innate lymphoid cells (ILC2s) [17]. These results were further confirmed in IL-33-deficient mice. Moreover, Th2 cell differentiation and eosinophilic lung inflammation were found to be impaired in intranasally challenged IL-33-deficient mice [18, 19]. IL-33 is the essential factor for severe allergic lung inflammation [20]. IL-33 signaling is required for causing airway eosinophilia and production of IL-5 as well as IL-13 from lung ILC2s following fungal allergen challenge *Alternaria alternate* [21]. IL-33 also seems to be essential for development of allergic rhinitis induced by ragweed pollen challenge as IL-33 knockout mice failed to induce ragweed pollen induce allergic rhinitis [22]. IL-33 is also an effective stimulator for skin ILC2s, and it is directly associated with skin inflammation and mouse model of atopic dermatitis [23].

The type 2 (ILC2) innate lymphoid cells localized in mucosal tissues (lung and intestine), adipose tissue and lymphoid organs (spleen, lymph node) are the major target cells of IL-33 [12, 24, 25]. These cells express high expression levels of ST2 and secretion of significant quantities of the type 2 cytokines IL-5 and IL-13, and pro-inflammatory IL-6 from ILC2. Further studies have shown that IL-33 not only activates mast cells, but also other immune cells, such as granulocytes, macrophages, NK and Th2 cells [26].

### Release of IL-33

**Fig. 1:**
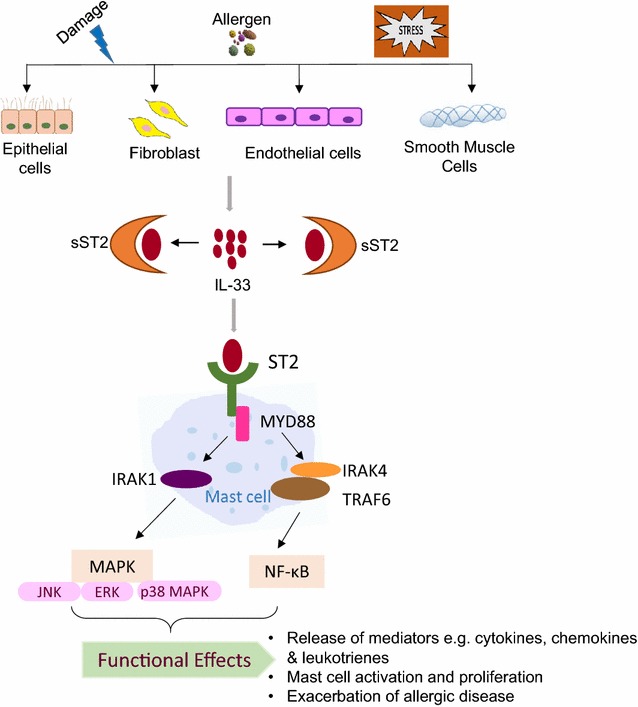
The IL-33/ST2 signaling pathway in mast cells. IL-33 is primarily expressed by different types of structural cells. Damage to these cells by external stimuli results in cell injury followed by release of IL-33. IL-33 can be neutralized by binding to soluble ST2 (sST2) or recognized by membrane bound ST2 receptor, which subsequently leads to activation of MyD88 dependent signaling pathways and the release of mast cell mediators that play important roles in the progression of allergic diseases

Interleukin-33 is stored in the nucleus and secreted upon necrosis or damage and released in response to cell injury, infection or mechanical damage [27, 28]. The high levels of constitutive IL-33 may act as a novel alarmin (intracellular alarm signal released after cell injury) to alert the immune system after endothelial or epithelial cell damage during trauma or infection (Fig. 1) [7].

The IL-1 family members do not possess signal peptide to release the proteins via endoplasmic reticulum and Golgi pathway [29]. Indeed, these interleukins are already translated and stored in the nucleus of the cells and released immediately during emergency conditions such as infection, injury or inflammation due to other stressors [30]. Extracellular IL-33 has also been detected in human blood and synovial fluids in pathological conditions [31], where cells have been damaged (during rheumatoid arthritis or infection with influenza virus, respectively) and in mouse peritoneal and bronchoalveolar lavage fluid [32]. However, in recent studies, it has been proposed that IL-33 may be released without cell damage and necrosis. Kouzaki et al. [33] found that exposing the human airway epithelial cells to *A. alternata* induces an acute extracellular danger signal, ATP, which releases IL-33 through activation of P2 purinergic receptors.

### Processing of IL-33

Interleukin-33 has been proposed to be a cytokine with dual function; it acts as a traditional cytokine through activation of the ST2L receptor complex as well as an intracellular nuclear factor with transcriptional regulatory properties. IL-33 shows the closest homology to IL-18 among the members of the IL-1 family of cytokines which are synthesized as full length pro-peptides. It was reported that IL-33 is synthesized as a 30 kDa peptide. It consists of N-terminal helix-turn-helix (HTH) motif responsible for nuclear translocation and chromatin binding [6] and an IL-1-like C-terminal domain, which is cleaved by caspase 1 to form an active 18 kDa mature peptide [28, 34, 35]. Later, it was confirmed that IL-33 protein contains the cleavage sites for caspase-3 and caspase-7 and it can be expressed and released by activated macrophages [36]. The processing of IL-33 by caspases results in its inactivation [28, 35]. IL-33 inactivation by caspases during apoptosis could be to prevent IL-33 release during programmed cell death, which does not require an inflammatory response.

Lingel et al. [37] studied the structure and interaction of IL-33 with its receptor ST2 and IL-1RAcP by X-ray crystallography as well as by NMR spectroscopy. Lefrancais et al. [38] demonstrated that mature form of IL-33 (after cleavage by mast cell proteases) are more potent than full length IL-33. During Inflammation, neutrophil-released proteases may regulate IL-33 activity. Mast cell serine proteases cleave the full-length IL-33 (IL-33_1–270_) and liberate active forms: IL-33_95–270_, IL-33_99–270_, and IL-33_109–270_. These cleaved IL-33 forms have 10 times greater potency than the full-length protein [38]. Roy et al. [39] reported that mast cell chymase but not tryptase cleaves IL-33 and results in increased bioactivity. This finding suggests that IL-33 activity could be exacerbated by the inflammatory environment. It has also been shown that serine proteases released by inflammatory cells play a critical role in the generation of super active forms of IL-33 and enhance immune response in asthma, rheumatoid arthritis, intestinal inflammation and other diseases [40, 41].

## Interleukin-33 signaling: involvement of the MyD88 and activation of MAP Kinases

The IL-33 mediated downstream signaling pathway is governed through ST2 and IL-1RAcP receptors. In an in vivo model, mice deficient either in IL-1RAcP or ST2 did not show an inflammatory reaction in response to IL-33 administration [42]. IL-33 binding by the ST2 receptor leads to the activation and recruitment of MyD88 adapter protein along with IL-1R-associated kinase1 (IRAK1), IRAK4 and TNFR-associated factor 6 (TRAF6) [5, 43]. This signaling cascade further leads to the activation of transcription factors such as NF-ĸB and MAP kinases and the production of inflammatory mediators (Fig. 1) [3]. MyD88 is crucial for several functional responses to IL-33 such as survival cytokine production and MCs proliferation [44, 45]. IL-33 treatment further leads to the activation of different kinases such as ERK1/2, p38MAPK, and JNK (Fig. 1) [5].

Interleukin-33 mediated signaling pathways further modulate MC functions. It has also been reported that IL-33 can activate MCs [46], and act directly on Th2 cells to increase secretion of Th2 cytokines such as IL-5 and IL-13 [5, 47]. Furthermore, IL-33 functions as a chemo-attractant for Th2 cells [48]. Research from several studies has indicated that IL-33 also acts as a potent activator of mast cells and basophils and reported to induce migration, maturation, adhesion, promote survival and the production of several pro-inflammatory cytokines in these cells [44, 49–51]. The IL-33 mediated downstream signaling pathway including functional aspects is shown in Table 2.

**Table 2:** IL-33 mediated downstream signaling cascade

Cytokine	Downstream signaling cascade	Cell type	Functional effect	References
IL-33	MyD88	BMMCs	Survival of BMMCs	[74]
IL-33	MyD88	BMMCs	Production of cytokine e.g. IL-6 and IL-13	[75]
IL-33	MyD88	BMMCs	Proliferation of mast cell	[45]
IL-33	MyD88	BMMCs	Release of IL-6 and IL-13	[74]
IL-33	MyD88	Intestine (mice)	Production of type 2 cytokine e.g. IL-4, IL-5 and IL-13	[76]
IL-33	MyD88	Lungs (mice)	Goblet cell hyperplasia	[77]
IL-33	p38 MAPK	BMMCs	Proliferation of mast cell	[45]
IL-33	p38 MAPK	BMMCs	IL-6 release	[46]
IL-33	p38 MAPK	BMMCs	IL-6 and IL-13 release	[74]
IL-33	JNK, ERK, p38 MAPK, NFκB	BMMCs	IL-6 and IL-13 release	[78]
IL-33	NF-κB and JNK1/2, ERK1/2, and p38 MAPK	BMMCs	Production of IL-4, IL-5, IL-13, CCL2, CCL17, and CCL24	[5]
IL-33	p38 MAPK	Human mast cell LAD2	IL-13 release	[79]
IL-33	p38MAPK	Human umbilical cord blood-derived mast cells (HUCBMCs)	IL-8 release	[49]

## Role of IL-33 in allergic disease

Interleukin-33 is considered to be linked to the development of several allergic diseases such as asthma and atopic dermatitis. IL-33 is also thought to accelerate Th17 cell-mediated airway inflammation via MCs [52]. Thus, it is evident from studies that IL-33 acts not only as a Th2-inducing cytokine, but also as a proinflammatory cytokine in various immune responses as do IL-1 and IL-18.

Asthma is characterized by chronic inflammation of the airways which is associated with variable airflow obstruction arising from various genetic and environmental factors. It involves the activation of MCs, Th2 cells, IgE producing B cells, basophils, eosinophils and lungs epithelial, smooth muscle cells and macrophages. Oshikawa et al. [53] and Hayakawa et al. [54] observed elevated levels of soluble ST2 as well as IL-33 mRNA in the serum and lung tissues, respectively in an ovalbumin (OVA)-induced murine asthma model of airway inflammation. Different advanced approaches, such as the use of anti-ST2 antibody (clone E310) [55, 56], anti-IL-33 antibody [57], or soluble ST2-Fc fusion protein [53] have been used to investigate the role of the ST2/IL-33 pathway in asthma models. Pre-treatment with these antibodies significantly inhibits airway inflammation and the Th2-associated responses. These antibodies also reduced IgE level in serum and the numbers of eosinophils in bronchoalveolar lavage in a murine model of allergic asthma. Kurowska et al. [47] detected IL-33 protein in the lungs of mice with OVA/alum-induced airway inflammation. IL-33 has been reported to also induce allergic bronchoconstriction through mast cell activation in mice [58]. IL-33 increases the expression of tryptophan hydroxylase 1, serotonin synthesis, and storage and thus results in airway obstruction in asthma [58].

Stolarski et al. [59] reported that IL-33 induces eosinophil mediated massive airway inflammation of the lung tissue and markedly elevated local concentrations of IL-5 and IL-13 and induced goblet cell hyperplasia in ova induced asthma model in mice. Lee et al. [60] investigated the role of anti-IL-33 antibodies and sST2 in the blockade of airway inflammation in a murine model of asthma and confirmed that both treatments were successful in reducing the total cell count and may serve as therapeutic agents for allergic asthma. Several studies have shown that IL-33 is expressed more abundantly in asthma patients than healthy individuals [47, 61]. Additionally, these results were also confirmed by elevated IL-33 expression in bronchial epithelial cells of asthma patients compared to healthy individuals by immuno-histochemical studies [59]. Role of IL-33 in mast cell activation and airway smooth muscle wound repair has also been reported [62] which suggests that IL-33 presents important target to modulate mast cell-airway smooth muscle (ASM) crosstalk in asthma.

Atopic dermatitis (AD) is a chronic inflammatory skin disease. Shimizu et al. [63] explored the association of AD with a polymorphism of the ST2 gene and suggested that the IL-33-ST2 axis plays a pivotal role in AD. Recently, Savinko et al. [64] and Meephansan et al. [65] reported the up-regulation of IL-33 in the epidermis and the infiltration of ST2-positive cells in the dermis of the skin lesion of AD patients. Imai et al. [66] reported that IL-33 from epidermal keratinocytes activates ILC2s in the skin and lymph nodes and stimulates the production of IL-5 from those cells to induce AD-like dermatitis with eosinophil infiltrates. On the basis of these observations IL-33 is considered as a unique danger alarmin and pathogenic driver in AD [67].

## Conclusions

Interleukin-33 is a unique cytokine that plays an essential role in regulating MC associated immune responses in allergic diseases. In the present scenario, the IL-33/ST2 pathway is being used as a novel therapeutic target for understanding the role of IL-33 in diseases associated with MCs. However, the elementary mechanisms of the release, expression, processing and regulation of IL‑33 in allergic diseases are not yet defined properly and may be crucial for the development of future therapeutic targets. Future studies are essential to recognize the biological and clinical significance of IL-33 in allergic diseases.

## Abbreviations

BMMCs: bone marrow-derived murine mast cell
ERK: extracellular signal-regulated kinases
HUCBMCs: human umbilical cord blood mast cells
IL-1RAcP: IL-1 receptor accessory protein
IL-1RL1: IL-1 receptor-like 1
ILCs: innate lymphoid cells
JNK: c-Jun N-terminal kinases
MyD88: myeloid differentiation primary response gene (88)
NF- κB: nuclear factor kappa-light-chain-enhancer of activated B cells
p38 MAPK: p38 mitogen-activated protein kinases
SGBS: Simpson-Golabi-Behmel syndrome
